# A proactive financial navigation intervention in patients with newly diagnosed gastric and gastroesophageal junction adenocarcinoma

**DOI:** 10.1007/s00520-024-08399-1

**Published:** 2024-02-24

**Authors:** Ari Bell-Brown, Talor Hopkins, Kate Watabayashi, Karen Overstreet, Anthony Leahy, Erin Bradshaw, Kathleen Gallagher, Jennifer Obenchain, Amber Padron, Beth Scott, Brooke Flores, Veena Shankaran

**Affiliations:** 1 Hutchinson Institute for Cancer Outcomes Research, Fred Hutchinson Cancer Center, 1100 Fairview Ave N., Mailstop M3-B232, Seattle, WA 98109 USA; 2 Consumer Education and Training Services, Seattle, WA USA; 3 Patient Advocate Foundation, Washington, DC USA; 4 Division of Medical Oncology, University of Washington School of Medicine, Seattle, WA USA

**Keywords:** Financial hardship, Financial navigation, Gastric cancer, Gastroesophageal junction cancer

## Abstract

**Purpose:**

Many cancer patients and caregivers experience financial hardship, leading to poor outcomes. Gastric and gastroesophageal junction (GEJ) cancer patients are particularly at risk for financial hardship given the intensity of treatment. This pilot randomized study among gastric/GEJ cancer patients and caregivers tested a proactive financial navigation (FN) intervention to obtain a signal of efficacy to inform a larger, more rigorous randomized study.

**Methods:**

We tested a 3-month proactive FN intervention among gastric/GEJ cancer patients and caregivers compared to usual care. Caregiver participation was optional. The primary endpoint was incidence of financial hardship, defined as follows: accrual of debt, income decline of ≥ 20%, or taking loans to pay for treatment. Data from participant surveys and documentation by partner organizations delivering the FN intervention was analyzed and outcomes were compared between study arms.

**Results:**

Nineteen patients and 12 caregivers consented. Primary FN resources provided included insurance navigation, budget planning, and help with out-of-pocket medical expenses. Usual care patients were more likely to experience financial hardship (50% vs 40%) and declines in quality of life (37.5% vs 0%) compared to intervention patients. Caregivers in both arms reported increased financial stress and poorer quality of life over the study period.

**Conclusions:**

Proactive financial navigation has potentially positive impacts on financial hardship and quality of life for cancer patients and more large-scale randomized interventions should be conducted to rigorously explore the impact of similar interventions. Interventions that have the potential to lessen caregiver financial stress and burden need further exploration.

**Trial registration:**

TRN: NCT03986502, June 14, 2019.

## Introduction

Financial hardship is a well-known struggle faced by cancer patients that encompasses a range of experiences such as loss of income, debt, and bankruptcy [1–6] and includes both material and psychological (e.g., anxiety about costs) aspects [7]. Cancer patients who experience financial hardship are at greater risk for treatment non-adherence, poorer quality of life, and worse survival [1–3, 5, 6]. Higher financial burden at the beginning of the disease can also result in more intensive hospital-based care, particularly at the end of life [8].

The financial consequences of cancer treatment extend beyond patients, affecting entire families and impacting caregivers’ sense of financial security, well-being, and ability to perform caregiving duties [8–15]. Informal caregivers are unpaid family members or friends who provide regular care or assistance to a friend or family member who has a health problem or disability [16]. Informal cancer caregivers often spend money on food, medications, and other patient needs in addition to taking time off work to provide support [17], with approximately 25% of cancer caregivers reporting taking 2 or more months of work leave to perform caregiving duties [12, 18–21]. Spouse or live-in partner caregivers are particularly vulnerable to financial hardship given the shared household income, assets, and expenses with patients [13, 14]. As a result of household financial impacts, caregivers that share household expenses may also experience poorer quality of life, depression, and higher caregiver burden [10, 22].

Gastric and gastroesophageal junction (GEJ) cancer patients and their caregivers are at particularly high risk of financial hardship and its psychosocial consequences given the intensity of treatment, impacts on employment, and additional costs related to transportation and food (e.g., supplements and special food preparation). New high-cost drugs approved for gastric and GEJ cancer treatment have contributed to longer survival but have also contributed to substantially higher cumulative treatment costs [23].

Despite the financial challenges faced by gastric and GEJ cancer patients and their caregivers, very few oncology clinics have specialists that provide assistance with medical costs or counsel families about management of assets, debts, and household expenses before financial challenges emerge [24, 25]. To address this gap, we have worked with two community organizations, Consumer Education and Training Services (CENTS)[26] and Patient Advocate Foundation (PAF) [27], to develop a program that offers: financial literacy resources, financial counseling, direct medical and healthcare cost and healthcare coverage assistance, and non-medical and indirect cost assistance to cancer patients and caregivers [25, 28]. We conducted a prospective pilot randomized trial of gastric and GEJ cancer patient-caregiver dyads at Fred Hutchinson Cancer Center (Fred Hutch) to explore the potential impact of proactive financial navigation on financial and clinical outcomes. This paper highlights the results of this pilot study.

## Methods

### Study population

Enrollment in this study was limited to patients 18 years or older who speak English as a primary language with stage I-IV gastric and GEJ adenocarcinoma within 6 months of their diagnosis and receiving systemic therapy. Patients enrolled in hospice care or with an Eastern Cooperative Oncology Group Performance Status of greater than 2 were excluded due to the unlikelihood that they could complete a 6-month study. There were no income or social requirements for participation in this study as we wanted to understand the impact of financial navigation on cancer patients across income levels. Patients were asked to designate a primary caregiver to participate with them; however, caregiver participation was optional. Caregivers were informal, unpaid caregivers who may or may not live in the same household as the patient. Caregiver inclusion criteria were broad, but caregivers were required to be 18 years or older and speak English. Patients were recruited from the Fred Hutch, an independent, nonprofit cancer care and research center in Seattle, Washington [29].

### Study design

**Fig. 1:**
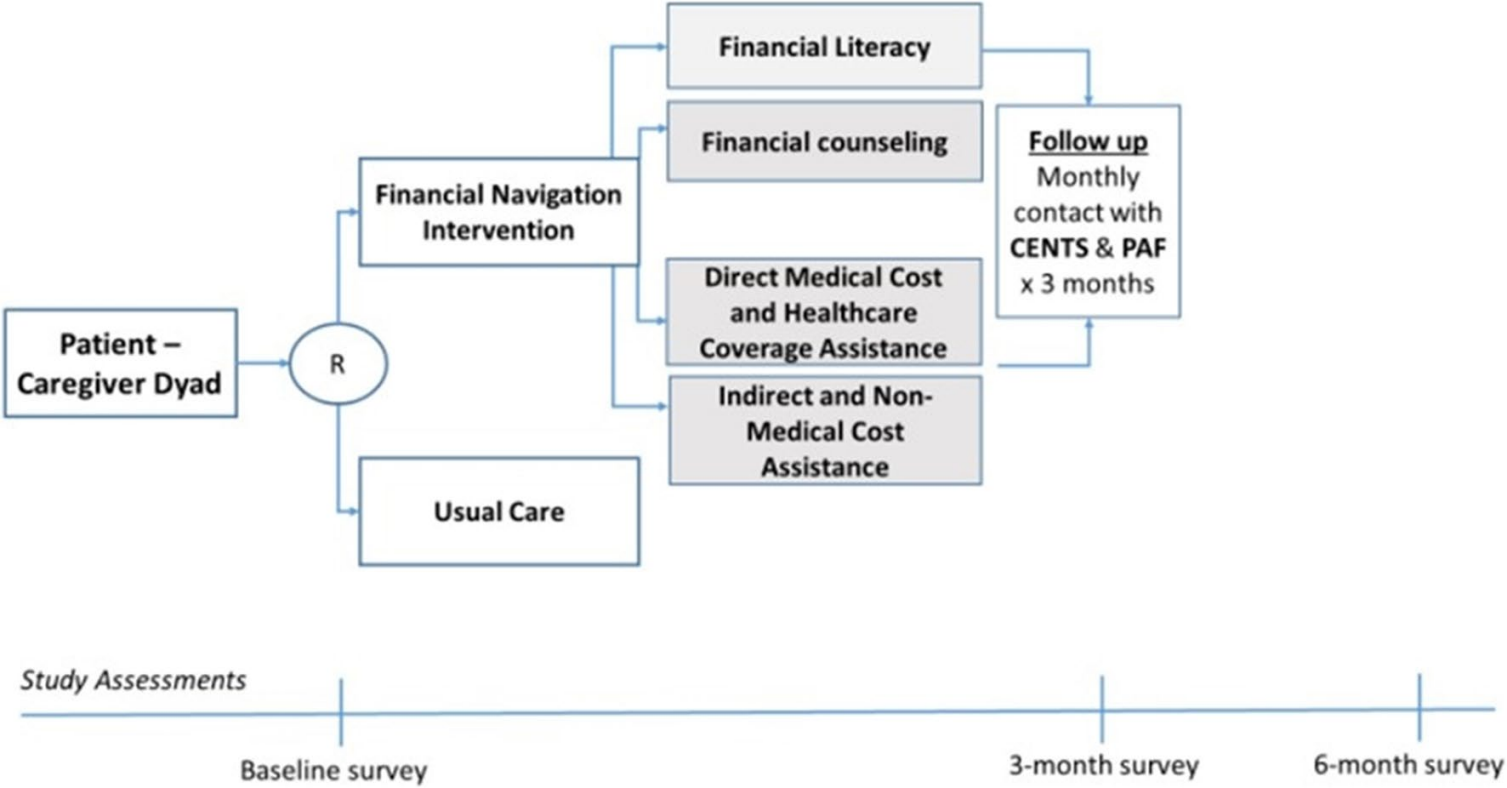
Study schema*.* Abbreviations: CENTS, Consumer Education Training Services; PAF, Patient Advocate Foundation

We conducted a pilot randomized study evaluating the impact of a 3-month proactive financial navigation intervention versus usual care. Goals of the pilot randomized design were to determine the feasibility of randomizing patient-caregiver dyads, track whether usual care participants who did not receive proactive financial navigation would still complete follow-up surveys, and to obtain a signal of efficacy with this intervention to inform a larger and more rigorous randomized study. Predictors and outcomes were assessed at baseline, 3 months, and 6 months of post enrollment. Figure 1 depicts the study schema.

#### Consent and enrollment procedures

Eligible patients were identified by their treating physician and confirmed via medical record review. Patients meeting the study eligibility criteria were approached by study staff using a recruitment letter sent via mail. Study staff then contacted patients over the phone to discuss participation and whether they had a caregiver who was interested in participating with them. Patients and caregivers were consented at the same time over the phone. All study procedures were reviewed and approved by the Fred Hutch Institutional Review Board.

Consented patients and caregivers were asked to complete a baseline survey via a secure Research Electronic Data Capture (REDCap) site assessing financial status, quality of life, and financial stress. All participants were provided with a financial navigation resource packet at enrollment containing a list of financial and cancer support resources, a list of transportation resources, and handouts about budgeting and how to address various financial and legal issues, especially in end-of-life situations. Following enrollment, study subjects in both arms received an email with a link to a series of six online financial literacy videos between 2 and 24 min in length [30] and were asked to view the videos within 14 days of enrollment.

Patient–caregiver dyads were randomized using a block randomization method, with randomization occurring at the patient level. Participants remained on study until voluntary withdrawal, death, or completion of all planned follow-up.

#### Usual care arm

After receiving the link to the videos, participants in the usual care arm followed normal clinic procedures for financial assistance and were able to utilize any available clinic or community-based financial resources. At Fred Hutch, usual care includes access to patient navigators who are able to provide assistance to patients and families with transportation, lodging, and social services and provide resources around financial, job-related, and insurance concerns [31].

#### Intervention arm

CENTS counselors and PAF case managers contacted subjects randomized to the intervention arm within 14 days of randomization to set up an initial session over phone or videoconference. After the initial meeting, enrolled participants were contacted monthly by CENTS and PAF for 3 months. CENTS is a Seattle-based non-profit organization that provides a variety of free educational programs to promote financial literacy among financially vulnerable groups throughout Western Washington, and their financial coaches have been trained to work directly with cancer patients and families to provide financial counseling and budget management [26]. PAF is a non-profit organization that provides professional needs navigation services to Americans with chronic, life-threatening, and debilitating illnesses, with case managers that serve as active liaisons between patient and insurers, employers, and/or creditors [27]. CENTS counselors and PAF case managers played complementary roles in delivering the main components of the intervention: financial counseling, direct medical cost and healthcare coverage assistance, and indirect and non-medical cost assistance.

#### Data collection and analysis

Prior to the start of participant enrollment, the Fred Hutch study team developed a secure REDCap site, accessible by study staff and CENTS counselors and PAF case managers. Surveys were collected from all participants at baseline, 3 months, and 6 months of post enrollment. CENTS and PAF documented encounters with intervention participants in REDCap that detailed the date, time, and duration of each call as well as any assistance given or instances where a financial issue could not be resolved. Patient demographics and clinical factors were collected from the electronic medical record.

The primary endpoint of the study was an incidence of household financial hardship, defined as self-report of one or more of the following within the 6-month study period: accrual of debt, income decline of ≥ 20%, or acquiring loans to pay for treatment [4, 32]. All study outcomes and their corresponding measures are listed in Table 1. Using a binary outcome measure (yes/no), we calculated the incidence of financial hardship within 6 months of enrollment among intervention versus usual care patients. To evaluate patient and caregiver quality of life, we used available scoring systems to determine composite Functional Assessment of Cancer Therapy- General (FACT-G) [33] (patient) and City of Hope Quality of Life (caregiver) scores at baseline, 3 months, and 6 months and looked at the proportion of participants in each arm who experienced improved, unchanged or worsened scores. We focused analysis on the change between baseline and 3 months, as 3 months marked the end of the active intervention period. A FACT-G score change of six points is considered clinically meaningful. Subjective financial distress was measured by the Comprehensive Score for Financial Toxicity-Patient Report Outcome Measure (COST-FACIT) tool and scored from 0 to 44, with a score of 26 or less considered financially distressed [34, 35]. Mean COST-FACIT scores at 3 and 6 months were calculated and compared between intervention and usual care participants. Documentation evaluated from CENTS counselors and PAF case managers was reviewed to characterize the types of assistance received by participants.

**Table 1:** Study outcomes and measures

Outcome	Measure (definition, data source, questionnaire, scale/subscale)
Patient outcomes	
Household financial hardship (primary endpoint)	Patient reports one of the following at the 3- or 6-month survey: accrual of debt, taking out loans to pay for cancer treatment, decline in household income by ≥ 20%
Patient quality of life	Composite score from the Functional Assessment of Cancer Therapy- General (FACT-G). A 6-point score change is considered clinically meaningful in US cancer populations
Patient and caregiver outcomes	
Subjective financial distress	Comprehensive Score for Financial Toxicity (COST-FACIT). 11-item measure developed for patients with advanced malignancies (scored 0–44)
Qualitative assessment of usual care and intervention	Usual care arm dyads will be surveyed about availability (or lack), access to, and use of financial assistance via the clinic and community. Intervention arm dyads will be surveyed about availability and use of financial assistance from the clinic, community, *and* navigation partners
Caregiver outcomes	
Caregiver quality of life	City of Hope Quality of Life Family Version, a well-validated tool with 37 items and 4 subscales. A change in score of 2 points per item is considered clinically meaningful
Caregiver burden	Social well-being subscale of the City of Hope Quality of Life Questionnaire

## Results

Recruitment started in January 2021 and was completed in April 2022, with 19 patients and 12 caregivers consenting to the study. Ten patients (8 of whom had participating caregivers) were randomized to the intervention and 9 patients (4 of whom had participating caregivers) were randomized to usual care. Median patient age was 67, 58% were male, and 89% had either Medicare or commercial insurance. Most participating caregivers were spouses (58%) or significant others (17%). Baseline patient and caregiver characteristics can be found in Table 2. One usual care caregiver did not fill out any surveys and is, therefore, not included in the results.

**Table 2:** Baseline patient and caregiver characteristics

Characteristic	Patients			Caregivers		
	Intervention (*N* = 10)	Usual care (*N* = 9)	*P*-value	Intervention (*N* = 8)	Usual care (*N* = 3)	*P*-value
Sex						
• Male	7 (70%)	4 (44%)	*P* = 0.25	2 (25%)	1 (33%)	*P* = 0.79
• Female	3 (30%)	5 (56%)	*P* = 0.25	6 (75%)	2 (67%)	*P* = 0.79
Race						
• White/Caucasian	10 (100%)	8 (89%)	*P* = 0.28	7 (87.5%)	3 (100%)	*P* = 0.52
• Mixed race	0	0		1 (12.5%)	0	*P* = 0.52
• Unknown	0	1 (11%)	*P* = 0.28	0	0	
• Ethnicity						
• Not Hispanic/Latino	9 (90%)	9 (100%)	*P* = 0.33	8 (100%)	3 (100%)	N/A
• Hispanic or Latino	1 (10%)	0	*P* = 0.33	0	0	
Age, median (range)	63 (45–78)	71 (34–79)	N/A	63 (47–68)	52 (34–63)	N/A
Insurance type				N/A	N/A	N/A
• Medicare	4 (40%)	5 (56%)	*P* = 0.48			
• Commercial	5 (50%)	3 (33%)	*P* = 0.45			
• Medicaid	1 (10%)	1 (11%)	*P* = 0.94			
ECOG performance score				N/A	N/A	N/A
• 0	2 (20%)	7 (78%)	*P* = 0.01*			
• 1	8 (80%)	2 (22%)	*P* = 0.01*			
Diagnosis				N/A	N/A	N/A
• GEJ cancer	8 (80%)	6 (67%)	*P* = 0.52			
• Gastric cancer	2 (20%)	3 (33%)	*P* = 0.52			
Cancer stage				N/A	N/A	N/A
• I or II	0	2 (22%)	*P* = 0.12			
• III	4 (40%)	4 (44%)	*P* = 0.86			
• IV	6 (60%)	3 (33%)	*P* = 0.24			
Annual household income, USD^a^						
• $0–$50,000	3 (33%)	2 (22%)	*P* = 0.60	4 (50%)	0	*P* = 0.12
• $50,001–$75,000	1 (11%)	1 (11%)	*P* = 1	0	0	
• $75,001–$100,000	1 (11%)	3 (33%)	*P* = 0.26	2 (25%)	0	*P* = 0.34
• > $100,000	4 (44%)	3 (33%)	*P* = 0.63	2 (25%)	3 (100%)	*P* = 0.03*
Marital status^a^				N/A	N/A	N/A
• Married/partnered	7 (78%)	5 (56%)	*P* = 0.32			
• Divorced	1 (11%)	2 (22%)	*P* = 0.53			
• Never married	1 (11%)	1 (11%)	*P* = 1			
• Widowed	0	1 (11%)	*P* = 0.31			
Relationship to patient	N/A	N/A	N/A			
• Spouse				5 (62.5%)	2 (67%)	*P* = 0.89
• Significant other				1 (12.5%)	1 (33%)	*P* = 0.43
• Friend				1 (12.5%)	0	*P* = 0.52
• Sister-in-law				1 (12.5%)	0	*P* = 0.52
Experienced financial hardship as a result of diagnosis^a^				N/A	N/A	N/A
• Yes	4 (44%)	3 (33%)	*P* = 0.63			
• No	5 (56%)	6 (67%)	*P* = 0.63			
Patient quality of life (mean composite FACT-G score)^a^	67.3	76.8	N/A	N/A	N/A	N/A
Financially distressed (COST-FACIT)^a,b^						
• Yes	5 (56%)	3 (33%)	*P* = 0.33	4 (50%)	1 (33%)	*P* = 0.62
• No	4 (44%)	6 (67%)	P = 0.33	4 (50%)	2 (67%)	*P* = 0.62
Change in employment as a result of diagnosis^a,c^						
• Yes	2 (22%)	2 (22%)	*P* = 1	1 (12.5%)	0	*P* = 0.52
• No	7 (78%)	7 (78%)	*P* = 1	7 (87.5%)	3 (100%)	*P* = 0.52
Caregiver Quality of Life (City of Hope Quality of Life), mean composite score	N/A	N/A	N/A	17	17.3	N/A
Caregiver burden (Social well-being subscale of City of Hope Quality of Life, mean composite score)	N/A	N/A	N/A	5.7	6.3	N/A

Abbreviations: *COST-FACIT* Comprehensive Score for Financial Toxicity, *ECOG* Eastern Cooperative Oncology Group, *FACT-G* Functional Assessment of Cancer Therapy-General, *GED* General Educational Development Test, *GEJ* gastroesophageal junction, *N/A* not applicable, *USD* United States dollar

*Results are significant

^a^These results reflect an *N* of 9 for patients in the intervention arm, as one intervention patient did not fill out the baseline survey

^b^Financial distress was calculated using the COST-FACIT measure, with a score of 26 or less being considered financially distressed

^c^Change in employment is defined as going from paid employment to disability, retirement, or a leave of absence

During the 3-month financial navigation period, CENTS and PAF provided intervention participants with resources for: interpreting insurance (*N* = 4), budget planning (*N* = 4), out of pocket medical expenses (*N* = 3), end of life arrangements (*N* = 2), living expenses (*N* = 2), medication payments (*N* = 2), insurance denials (*N* = 2), emotional support (*N* = 2), disability benefits (*N* = 2), charity care (*N* = 1), cost of insurance premium (*N* = 1), employment protections (*N* = 1), transportation (*N* = 1), and nutritional support (*N* = 1). While all ten patients in the intervention arm had at least one interaction with CENTS and PAF, two participants with reported household incomes of over $100,000 declined needing any resources from either organization. Patients in the intervention arm with a household income of $50,000 or less (*N* = 3) were more likely to require assistance with both medical and non-medical/indirect costs related to their diagnosis compared to patients with household incomes greater than $50,000 who mainly focused their FN sessions on insurance navigation, budget planning, and end of life arrangements. Only one usual care arm participant reported accessing financial assistance through a community organization, Wellness House [36], over the course of the study period.

Table 3 shows patient results. In the 6 months after enrollment to the study, four households in the usual care arm developed financial hardship and two households in the intervention arm developed financial hardship. While there was not an overall difference in mean quality of life between intervention arm patients and usual care patients, more usual care patients (*N* = 3) saw a clinically meaningful dip (6-points or more) in their FACT-G score compared with intervention arm patients (*N* = 0) at 3 months. Subjective financial distress as measured by the COST-FACIT was similar between arms, although more patients in the intervention arm were financially distressed at baseline (Table 2). Across arms, 9 out of 19 (47%) patients experienced an insurance denial over the course of the study.

**Table 3:** Patient outcomes

Outcome	Intervention arm^a^	Usual care^b^	*P*-value
Development of financial hardship	2/5 (40%)	4/8 (50%)	*P* = 0.7263
Improved quality of life^c^ at 3 months	2/5 (40%)	3/8 (37.5%)	*P* = 0.9283
Unchanged quality of life^c^ at 3 months	3/5 (60%)	2/8 (25%)	*P* = 0.2077
Decreased quality of life^c^ at 3 months	0/5 (0%)	3/8 (37.5%)	*P* = 0.1188
Financially distressed^d^ at 3 months	1/5 (20%)	1/8 (12.5%)	*P* = 0.7188
Took extended paid time off, unpaid time off, or reduced work hours	4/9 (44%)	5/9 (56%)	*P* = 0.6101
Faced insurance denials	5/9 (56%)	4/9 (44%)	*P* = 0.6101
Sold or refinanced home	1/9 (11%)	2/9 (22%)	*P* = 0.5287

Abbreviations: *COST-FACIT* Comprehensive Score for Financial Toxicity, *FACT-G* Functional Assessment of Cancer Therapy-General

^a^Five intervention arm patients filled out follow-up surveys and 9 filled out the baseline survey

^b^Eight usual care arm patients filled out follow-up surveys and 9 filled out the baseline survey

^c^Patient quality of life was calculated using composite FACT-G scores, with a 6-point score change being clinically meaningful. 3-month results included as this was the end of the active intervention period

^d^A COST-FACIT score of 26 or less is considered financially distressed. 3-month results included as this was the end of the active intervention period

While caregiver quality of life and subjective financial distress did not differ between arms suggesting the intervention did not impact caregiver outcomes, most caregivers (*N* = 7, 64%) across arms stated that being a caregiver has increased their financial worry. Caregivers reported dipping into their savings and other accounts and cutting back spending in a variety of areas because of providing care. Caregiver outcomes are shown in Table 4. Despite the financial concerns, all caregivers reported on their surveys that they had adequate resources in the past 4 weeks prior to filling out the survey to meet their family’s daily needs most or all of the time.

**Table 4:** Caregiver outcomes^a^ (*N* = 11 caregivers)

Outcome	*N* (%)
Cut back on work hours	7 (64%)
Took unpaid leave or FMLA	4 (36%)
Used sick or vacation time	6 (54%)
Took another job or worked extra hours	1 (9%)
Dipped into savings accounts	5 (45%)
Dipped into retirement accounts	1 (9%)
Took out loans or increased credit card debt	2 (18%)
Declared bankruptcy	1 (9%)
Cut back on spending for vacation/travel	4 (36%)
Cut back on spending for groceries	3 (27%)
Cut back on health or dental care	3 (27%)
Cut back on home maintenance	1 (9%)
Cut back on other necessities	3 (27%)
Financially distressed (mean COST-FACIT^b^) (*N* = 7 respondents)	4 (57%)
Change in quality of life^c^	− 1.9
Change in caregiver burden^d^	− 0.4

Abbreviations: *COST-FACIT* Comprehensive Score for Financial Toxicity, *FMLA* Family Medical Leave Act

^a^Caregiver outcomes are reported as combined outcomes over the course of the study period among both intervention and usual care arm caregivers

^b^Number of caregivers reporting financial distress after baseline. A COST-FACIT score of 26 or less is considered financially distressed

^c^Change in mean City of Hope Quality of Life score, a 2-point change is clinically meaningful

^d^Change in mean City of Hope Quality of Life- Social well-being subscale, a 2-point change is clinically meaningful

## Discussion

This study tested a financial navigation and financial literacy program in a population of gastric and GEJ cancer patients. This program has also been piloted among patients with nonmetastatic solid tumors as well as patients with any-stage solid tumor diagnosis [28, 37]; however, this is the first pilot randomized study conducted. Although the number of participants was low, the households in the intervention arm of our study faced slightly lower rates of financial hardship (*N* = 2 intervention households (40%) vs *N* = 4 usual care households (50%)) 6 months following delivery of the financial navigation program, despite the average income of usual care participants being slightly higher than in the intervention arm. Additionally, more usual care patients experienced decreased quality of life at 3 months, the end of the financial navigation period, compared with intervention arm patients (37.5% of patients vs 0%, respectively). These modest yet intriguing findings hint at proactive financial navigation having potentially positive impacts on both financial hardship as well as quality of life for cancer patients and support the development of larger, randomized efficacy studies to test this hypothesis.

Despite the high-income levels of participants, most intervention patients still requested assistance from CENTS and PAF in a variety of areas, with higher income participants (> $50,000) receiving more support in how to interpret their insurance benefits, handling insurance denials, medication payments, and end of life arrangements. While this was a well-insured group, several reported facing insurance denials in the 6-month period of the study. Reported insurance denials were for immunotherapy prescriptions, imaging, and supplements such as potassium powder.

Caregiver outcomes did not vary much between arms, but the financial and emotional burden of being a caregiver was commonly reported across both arms. Caregivers reported lower quality of life than patients through all three surveys and the majority of caregivers (64%) reported that becoming a caregiver had increased their financial worry. Many caregivers reported changes in work hours and spending patterns as a result of providing care to the patient. This data reminds us that it is important to include caregivers in research on the financial hardship of a cancer diagnosis. Further interventions targeting both patient and caregiver financial hardship should be explored.

Previous pilot studies testing this proactive financial navigation and literacy program across multiple cancer types have shown the program to be feasible for cancer patients and their caregivers to participate in and have shown some signals of reducing cancer-related financial hardship, both in material and psychosocial outcomes [28, 37]. In recognition that the program would benefit from being tested in a larger scale intervention across several types of health systems to determine whether financial navigation programs should be incorporated as a standard part of cancer care delivery, the study team has been funded through the National Cancer Institute (NCI) to test this program in a large, randomized trial across National Cancer Institute Community Oncology Research Program (NCORP) sites. A Randomized Trial Addressing Cancer-Related Financial Hardship Through Delivery of a Proactive Financial Navigation Intervention (CREDIT, NCT04960787) was launched in July 2021 and is actively recruiting patient-spouse/partner caregiver dyads across NCORP sites to receive 6 months of proactive financial navigation post cancer diagnosis requiring systemic treatment. This large-scale trial will provide robust evidence on whether financial navigation for patients planning to receive anti-cancer treatment decreases the risk of material household financial hardship among patients with newly diagnosed or recurrent metastatic solid tumor, or a newly diagnosed hematologic malignancy, and their spouse or partner caregivers.

Our study has several limitations to note. The first limitation is the small number of participants. The study was first launched at the beginning of the coronavirus (COVID-19) pandemic, causing the study team to have to pivot to remote recruitment among an already limited number of potential participants due to the less common nature of gastric and GEJ cancer diagnoses. We observed a higher rate of participant decline compared to other pilot studies due to the overwhelming nature of a gastric/GEJ cancer diagnosis, which is very often diagnosed at later stages and has poorer outcomes compared with other solid tumors. Additionally, this study was conducted at a single institution that serves a largely white, higher income, and insured population. These findings therefore may not be generalizable to a more socioeconomically and racially diverse population. Despite these limitations, we feel that these findings in partnership with our other pilot studies suggest there is value in conducting a large-scale randomized trial with a longer follow-up period of proactive financial navigation for patients and caregivers in more diverse healthcare settings that can assess outcomes among younger, financially fragile, and lower-income patients and households who may be most at risk for financial hardship after a cancer diagnosis.

## Conclusions

In a pilot randomized study of a proactive financial navigation and literacy program, gastric and GEJ cancer patients and their caregivers were less likely to experience financial hardship at 6 months of post enrollment and also less likely to experience a decrease in quality of life at 3 months of post enrollment (end of financial navigation period) if they received navigation. More large-scale interventions are necessary to understand the impact of proactive financial navigation on the development of financial hardship from a cancer diagnosis. Additionally, caregiver financial burden should be further explored and interventions tested as caregivers face significant financial and emotional distress as a result of providing care.

## Data Availability

The raw data that support the findings of this study are not openly available due to reasons of sensitivity and are available from the corresponding author upon reasonable request.
